# Taste aversion learning during successive negative contrast

**DOI:** 10.3758/s13420-024-00626-3

**Published:** 2024-02-08

**Authors:** Robert A. Boakes, Connie Badolato, Simone Rehn

**Affiliations:** School of Psychology (A18), University of Sydney, Sydney, NSW 2006 Australia

**Keywords:** Sucrose, Saccharin, Negative contrast, Taste aversion, Rats

## Abstract

Previous experiments found that acceptance of saccharin by rats was reduced if they had prior experience of sucrose or some other highly palatable solution. This study tested whether such successive negative contrast (SNC) effects involve acquisition of an aversion to the new taste. In three experiments, rats were switched from sucrose exposure in Stage 1 to a less palatable solution containing a new taste in Stage 2. In Experiments 1 and 2, a novel flavor was added to a saccharin solution at the start of Stage 2. In Experiment 1, preference tests revealed a weak aversion to the added vanilla flavor in the *Suc-Sacch* group, while in Experiment 2 an aversion was found in the *Suc-Sacch* group to the salty flavor that was used, compared with controls given access either saccharin or water in Stage 1. In Experiment 3, the *Suc-Quin* group, given quinine solution in Stage 2, displayed a greater aversion to quinine than a *Water-Quin* control group. These results support the suggestion that taste aversion learning plays a role in the initial suppression of intakes in a qualitative consummatory SNC effect. However, in the light of other evidence, it seems that the unusual persistence of successive negative contrast when rats are switched from sucrose to saccharin is not due to a long-lasting reduction in the value of saccharin.

Successive negative contrast (SNC) normally refers to an abrupt decrease in performance when a less-preferred outcome is unexpectedly substituted for a previously available, highly preferred outcome (Flaherty, 1996). In the classic study of this phenomenon, a group of rats was first trained to run down a single runway to a goal box in which they found 256 ‘unit incentives’; when only 16 units were made available, running speeds decreased abruptly and then recovered to the level of controls given 16 units throughout (Crespi, 1942). SNC has been documented in a variety of other preparations and in a variety of mammalian species; however, it has proved difficult to obtain the effect in other vertebrates (Papini, 2003). Nonetheless, at least one study has demonstrated SNC in a bird species, the European starling (Freidin et al., 2009).

A procedure used extensively by Flaherty and his collaborators to study SNC measures rates of licking by rats for palatable solutions. For example, when a group of rats was given access to a 32% sucrose solution over a number of brief daily sessions, they licked at a high rate—one much higher than a group given access to 4% sucrose solution; when the 32% rats were switched to the 4% solution, the 32-4 group, their lick rate decreased to a level less than half that of the rats given 4% from the start, the ‘unshifted’ 4-4 group (Flaherty & Largen, 1975). Using this method, similar results were obtained when suitable concentrations of saccharin solutions were used (Flaherty & Rowan, 1986).

In a recent study, we documented what appeared to be a further example of SNC. To examine recovery from impairments induced by high intakes of 10% sucrose solution, we ran two translational experiments that contained groups switched from sucrose to saccharin; in both experiments, intakes of saccharin remained puzzlingly low across extended testing (Kendig et al., 2018). Subsequent experiments confirmed that this low acceptance of saccharin occurred as a result of prior experience of more palatable solutions. In addition to 10% sucrose, prior exposure to 10% solutions of glucose or of some types of maltodextrin was also found to reduce subsequent acceptance of a saccharin solution, in comparison with rats given only water prior to the introduction of saccharin (Boakes et al., 2020).

Although these results suggested that reduced acceptance of saccharin was a form of SNC, the evidence was incomplete. First, the above experiments did not include a group given saccharin from the outset that would correspond to the unshifted 4-4 control group in the example described earlier that used changes in sucrose concentration (Flaherty & Largen, 1975). Thus, a difference in acceptance of saccharin between water-preexposed and sucrose-preexposed groups could reflect either a SNC effect in the sucrose group or a successive positive contrast (SPC) effect in the water group, or a combination of both. Consequently, the first two experiments reported here contained a saccharin-to-saccharin (*Sacch-Sacch*) control group.

A second obstacle to concluding that the low acceptance of saccharin was an example of SNC was its persistence; SNC is usually transient. For example, Pellegrini et al. (2004) used a procedure in which rats were given 20 daily trials and in each trial a drinking tube was inserted into their conditioning chamber for 5 min; the dependent variable was ‘goal tracking time’—namely, the time a rat spent in contact with the tube. The key group (32-4) was first given 32% sucrose and then switched to 4% sucrose. The switch produced an immediate decrease in mean goal tracking time from around 250 s to 60 s in the first postswitch session. However, these times rapidly increased over the next 10 sessions until they were no longer different from those of the control (4-4) group. The speed of this recovery was similar to that of running speeds in Crespi’s (1942) study and to that observed in the majority of experiments on SNC (Flaherty, 1996).

There are at least two major differences between the studies in which we have found a reduction in saccharin acceptance and the methods used in most SNC studies. The first is the use of relatively short discrete trials in most SNC studies as opposed to the 24-h intakes of saccharin we have previously recorded. To approximate more closely to the methods used in typical SNC experiments, the present experiments provided access to the solutions in relatively short sessions held in separate drinking chambers.

The second difference was the use of *quantitative* shifts in most SNC studies (e.g., a reduction in the number of unit-incentives or concentration of sucrose) as opposed to *qualitative* shifts, as in the change of the taste from sucrose to saccharin. Interestingly, the last published experiment in the long series of SNC studies reported from Flaherty’s laboratory unusually involved a qualitative shift from a highly palatable mixture of glucose and saccharin to 2% glucose alone; the resultant low acceptance of the glucose solution, compared with controls, persisted over eight postshift sessions (Mitchell & Flaherty, 2005; see Fig. 1). Another study that used a qualitative shift was the experiment involving starlings cited earlier. Here, the shift was from mealworms to the less preferred ‘turkey crumbs.’ This produced a typical SNC effect that progressively extinguished over nine sessions (Freidin et al., 2009; see Fig. 1). It may also be noted that the first report of an SNC effect, based on time rats took to traverse a complicated maze, also involved a qualitative change, in this case from highly valued bran mash to sunflower seeds (Elliott, 1928; see also Pellegrini & Mustaca, 2000).

**Fig. 1:**
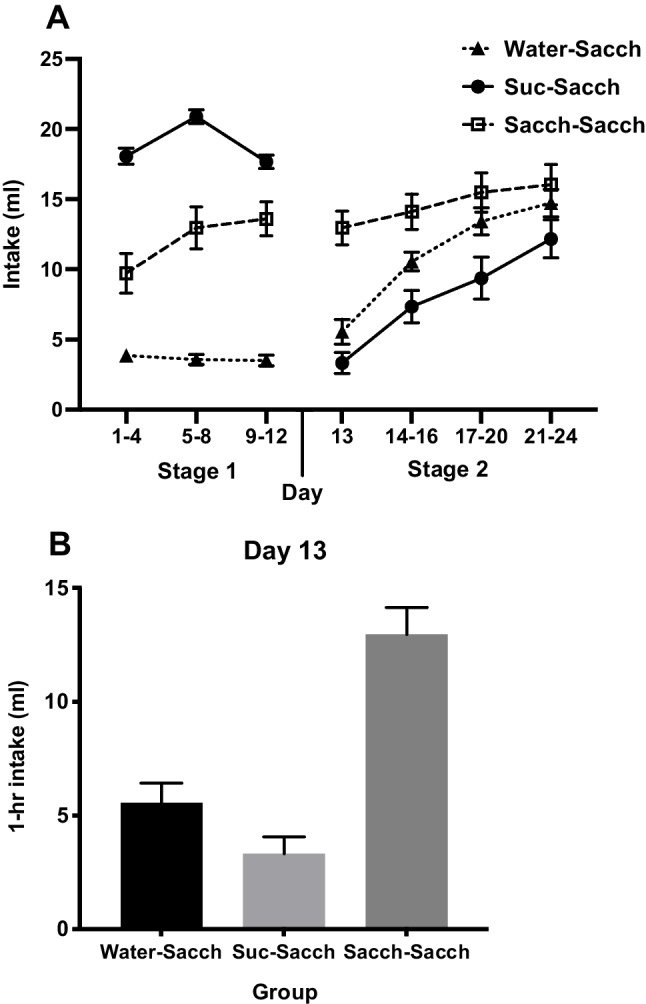
Experiment 1. **A** Mean intakes (±*SEM*) in Stage 1, in which groups were given either 10% sucrose (*n* = 12), 0.4% saccharin (*n* = 10) or water (*n* = 9) and in Stage 2, in which all groups were given 0.4% saccharin. The latter solution was flavored with 1% vanilla for the first session of Stage 2 (Day 13). **B** Mean intake (+*SEM*) of vanilla-flavored saccharin on Day 13

The main aim of the present study was to test the possibility that, when a downshift from a highly palatable solution involves the introduction of a new taste, an aversion is acquired to this taste. We propose that such a conditioned aversion is based on an association between the new taste and the negative emotional response—frustration—to the sudden absence of the expected highly valued incentive. The approach to testing the taste-aversion hypothesis adopted in the present three experiments was to introduce novel tastes at the start of the downshift stage and assess liking for the taste in a subsequent two-bottle preference test.

In a parallel study we assessed this possibility by measuring the size of lick clusters when rats were given saccharin in the down-shift stage. The results provided some support for our taste-aversion hypothesis, in that lick clusters were initially smaller in a sucrose-to-saccharin group than in various controls that were also given saccharin in the postshift stage. However, this reduction in the palatability of saccharin was relatively short-lived whereas the reduction in intake was persistent, as previously found (Rehn et al., 2023).

## Experiment 1

The main aim of this experiment was to detect in rats switched from sucrose to saccharin possible conditioning of an aversion to a vanilla flavor that was added to the saccharin solution for the first saccharin session.

Consistent with previous evidence that female albino rats are more accepting of 0.4% saccharin solutions than are males (e.g., Dess, 1993), our previous study suggested that reduced acceptance of saccharin following sucrose exposure was likely to persist longer in females than in males (Boakes et al., 2020). Partly for this reason, females were used in this and the following experiments.

Preference for saccharin at the end of the experiment was assessed relative to maltodextrin. Previously, we have assessed saccharin relative to 2% sucrose. However, group differences in such a test are ambiguous; in particular, 2% sucrose in the later test can be more palatable to a group given 10% sucrose in Stage 1 than to the nonsucrose groups and thus the saccharin versus 2% sucrose test may be misleading in suggesting an aversion to saccharin (Rehn et al., 2023). A maltodextrin solution was chosen because, although isocaloric with a comparable sucrose solution, it differs in taste (Ramirez, 1994; Sclafani, 1991). Furthermore, previous unpublished preference tests in our laboratory found that a 4% maltodextrin solution (of the type used in the present experiment) is about as palatable to rats as a 0.4% saccharin solution.

As shown in Table 1A, there were three groups—*Suc-Sacch*, *Sacch-Sacch* and *Water-Sacch*—that differed only in terms of the solution they were given in Stage 1. The *Water-Sacch* group was included as a control for neophobia towards the saccharin solution.

**Table 1:** Summary of the design of the three experiments

A. Experiment 1					
Groups			Stage 1 (12 sessions)	Stage 2 (12 sessions)	Preference tests
Suc-Sacch			10% sucrose	*Session 1*: Vanilla + 0.4% saccharin, *Sessions 2–12*: 0.4% saccharin only	Vanilla *vs* water, and Saccharin *vs* maltodextrin
Sacch-Sacch			0.4% saccharin		
Water-Sacch			Water		
B. Experiment 2					
Groups	Stage 1 (12 × 30-min sessions)	Stage 2 (12 × 30-min sessions)	Preference tests	Stage 3 (Extinction)	Preference test
Suc-Sacch	10% sucrose	*Sessions 1-2*: NaCl + 0.4% saccharin, *Sessions 3–12*: saccharin only	NaCl *vs* water, & Saccharin *vs* maltodextrin	NaCl or water	NaCl *vs* water
Sacch-Sacch	0.4% saccharin				
C. Experiment 3					
Groups		Stage 1 (12 × 30-min sessions)	Stage 2 (5 sessions)	Stage 3 (12 × 30-min sessions)	Preference test #2
Suc-Quin		10% sucrose	*Sessions 1–2*: Quinine, *Sessions 3–4*: Water, then Quinine *vs* water preference test #1	Quinine	Quinine *vs* water
Suc-Water				Water	
Water-Quin		Water		Quinine	
Water-Water				Water	

### Method

#### Subjects

Thirty-six female Sprague-Dawley rats, aged 8 weeks on arrival, were purchased from the Animal Resources Centre, Perth, Western Australia. They were group housed, with four rats to a cage, and were maintained under reverse-light-cycle conditions (lights off at 10:00 hr; on at 22:00 hr). Rats were given unrestricted access to chow throughout the experiment. As detailed below, prior to some sessions, water bottles were removed beforehand; otherwise, access to water was also unrestricted. Rats were handled for 5 days prior to the start of the experiment, at which point the average weight was 210 g (range: 192–225 g). They were weighed every 8 days, and cage bedding was changed twice a week.

#### Apparatus and solutions

Two types of drinking chamber were used for the presentation of experimental fluids. These were located adjacent to each other in a laboratory adjoining the colony room, and each rat was always placed in the same chamber throughout the experiment. The first set of chambers comprised 18 acrylic cages, measuring 23 × 35 × 19 cm, with steel wire lids and paper chip bedding covering the floor. The other set comprised 18 steel wire cages, measuring 19.5 × 28 × 18 cm. Allocation to these two types of drinking cages was counterbalanced across groups, with six animals from each group in each type of chamber.

Fluids were presented in plastic bottles with stainless-steel ball-bearing spouts that could be inserted between wires of the cage lids. The bottles were weighed to the nearest 0.1 g before and after each session to measure consumption. The target flavor was a solution of 1% (v/v) imitation vanilla (Queen brand). A 10% (w/v) sucrose (commercial white sugar) solution was used during Stage 1. The 0.4% (w/v) saccharin solution was prepared from saccharin sodium salt hydrate (Sigma S-1002). A 4% (w/v) solution of maltodextrin (Myopure Maltodextrin DE17, www.myopure.com.au) was used in the saccharin preference test.

#### Procedure

There were six sessions a week starting at 1500 hr. All rats were first given access to water in their drinking chambers to habituate them to drinking in this context. On the first day, 30-min access was given, 60-min on the second day, and 90-min access on the third. Rats were then allocated to three groups (*n* = 12) matched for body weight.

On each day of the 12-day Stage 1, rats were transferred to their drinking chambers and given access to either 10% sucrose (*Suc-Sacch* group), 0.4% saccharin (*Sacch-Sacch* group) or water (*Water-Sacch* group). For the first 6 days these sessions lasted 2 h, and for the last 6 days they lasted 1 h. All 12 sessions in Stage 2 lasted 1 h. In the first session of Stage 2, all rats were given access to vanilla-flavored saccharin solution. On subsequent days they were given unflavored saccharin solution.

To familiarize rats with the two-bottle test procedure, they received 15-min access to two bottles containing water on two consecutive days. Intakes from left and right bottles were calculated to assess potential side preferences; any rat drinking >80% to the same side on both days would be considered to show a strong position preference, but this did not occur for any rat. During this training and the tests that followed, if a session was also to be conducted the following day, on return to the home cages, water bottles were replaced for 30 min and then removed for overnight water deprivation. Two vanilla preference tests were conducted. On the first, a solution of 1% vanilla in water was on the left and unflavored water on the right; these positions were reversed for the second test session. Prior to the saccharin preference tests, rats were given one-bottle access to 4% maltodextrin in a single 1-h session to familiarize them with this solution. Over the following 2 days, the rats were given 15-min two-bottle tests between 0.4% saccharin and 4% maltodextrin, with saccharin on the left for the first session and positions reversed for the second test.

These procedures and those used in the remaining two experiments were approved by the University of Sydney Animal Ethics committee under Project No: 2021/1929.

#### Data analysis

Fluid intakes of Stages 1 and 2 were averaged for each of three successive 4-day blocks and analyzed with 3 × (3) Group × Block mixed analyses of variance (ANOVAs). Mixed 3 × (2) Group × Test ANOVAs were applied to preference test ratios. Significant group effects were examined with uncorrected pairwise comparisons. Additionally, one-way ANOVAs were applied to average intakes of each block in Stage 2, as well as the first vanilla and saccharin preference tests. Preference data from the two-bottle tests were analyzed with planned contrasts which compared (1) *Suc-Sacch* versus *Sacch-Sacch* (to assess flavor aversion in the *Sucrose* group) and (2) *Suc-Sacch*+*Sacch-Sacch* versus *Water-Sacch* (to assess flavor preference in the *Water*-*Sacch* group). Effects were considered significant when *p* < .05.

## Results

The intake measures for three rats from the *Water-Sacch* group and two rats from the *Sacch-Sacch* group were unreliable; these rats regularly rattled their bottles causing spillage and occasionally empty bottles. Their data were excluded from all the analyses reported here.

Fluid intakes of both Stage 1 and Stage 2, averaged over four-session blocks, are shown in Fig. 1A. As expected, and as this figure suggests, in Stage 1 intakes of sucrose were greater than intakes of saccharin and these were in turn greater than intakes of water. This description was confirmed by a mixed ANOVA that revealed a main effect of group, *F*(2, 28) = 104.57, *p* < .001, η_p_^2^ = .88, and of block, *F*(2, 56) = 9.13, *p* < .001, η_p_^2^ = .25, and an interaction, *F*(4, 56) = 7.67, *p* < .001, η_p_^2^ = .35. Stage 1 intakes in the *Suc-Sacch* group were greater than intakes in both the *Sacch-Sacch* and *Water-Sacch* groups, *p*s < .001, and the *Sacch-Sacch* group consumed more than the *Water-Sacch* group,* p* < .001.

Intakes of vanilla-flavored saccharin in the first session of Stage 2 are shown in Fig. 1B. A one-way ANOVA of these intakes confirmed a significant difference between groups, *F*(2, 28) = 29.06, *p* < .001, η_p_^2^ = .68. Pairwise comparisons found the *Sacch-Sacch* group consumed more than both the *Suc-Sacch* and *Water-Sacch* groups, *p*s < .001, whereas no significant difference between the *Suc-Sacch* and *Water-Sacch* groups was found, *p* > .10.

For the remainder of Stage 2 intakes of unflavored saccharin were averaged across Days 14–16, and then across the next two four-session blocks (see Fig. 1A). A mixed ANOVA confirmed a main effect of group, *F*(2, 28) = 5.89, *p* = .007, η_p_^2^ = .30, and of block,* F*(2, 56) = 37.49, *p* < .001, η_p_^2^ = .57, as well as an interaction, *F*(4, 56) = 2.72, *p* = .038, η_p_^2^ = .16. Averaged across blocks, intakes in the *Sacch-Sacch* group were higher than in the *Suc-Sacch* group, *p* =.002. The *Water-Sacch* group drank slightly more than the *Suc-Sacch* but this was marginally significant, *p* = .05 and there was no difference detected between the *Sacch-Sacch* and *Water-Sacch* groups, *p* = .37.

Follow-up pairwise comparisons for the Group x Block interaction found that the difference between *Suc-Sacch* and *Sacch-Sacch* groups was significant across first (*p* < .001), second (*p* = .003) and third (*p* = .042) blocks of Stage 2. The *Sacch-Sacch* versus *Water-Sacch* comparison also reflected increasing saccharin intake in the *Water-Sacch* group; *Sacch-Sacch* rats had greater intakes than *Water-Sacch* rats in the first block (*p* = .04), but this was no longer significant in the second (*p* = .31) and third (*p* = .51) blocks. Finally, a difference between the *Suc-Sacch* and *Water-Sacch* groups was only found in the second block where *Water-Sacch* rats had higher intakes than *Suc-Sacch* rats, *p* = .04. This difference was marginally significant in the first (*p* = .05) block but disappeared in the third block (*p* = .18).

**Fig. 2:**
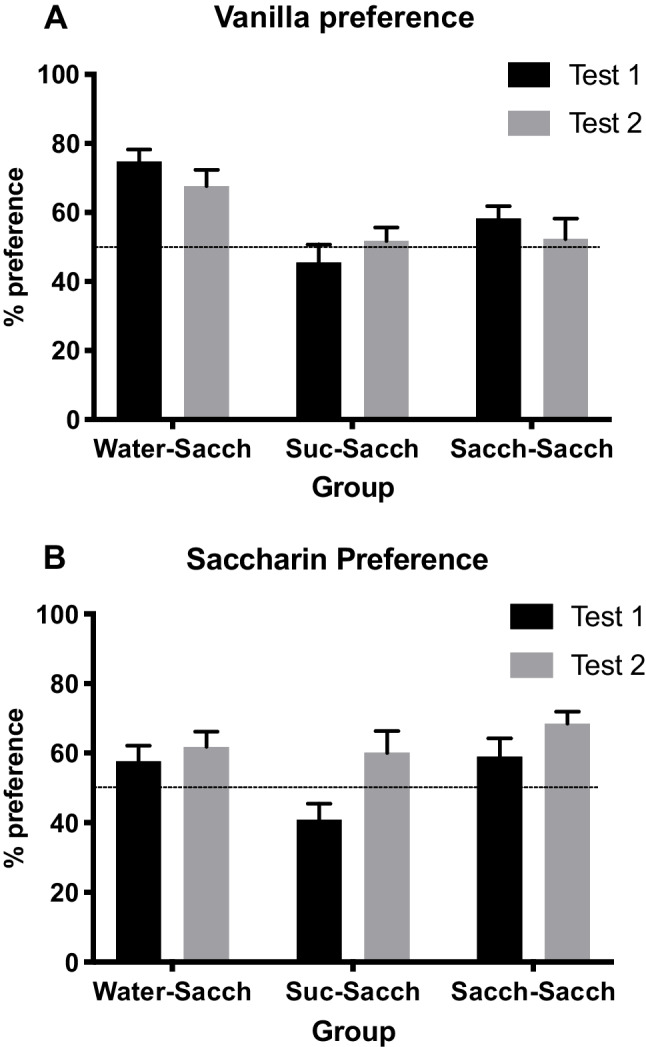
Experiment 1. Mean preference ratios (+*SEM*): **A** Vanilla-flavored water versus unflavored water. **B** 0.4% saccharin versus 4% maltodextrin

Vanilla preferences are shown in Fig. 2A. A mixed ANOVA of these data found a main effect of group, *F*(2, 28) = 11.68, *p* < .001, η_p_^2^ = .46, but no main effect of test or interaction, (*p*s > .10). The first planned contrast examining the Group effect found vanilla preferences in the *Sacch-Sacch* (*M* = 55.2%) and *Suc-Sacch* (*M* = 48.6%) groups did not differ, *p* > .10, indicating no vanilla aversion in the *Suc-Sacch* group, when averaged across both tests. The second contrast compared preferences in the *Water-Sacch* group (*M* = 71.2%) with those of the *Sacch-Sacch* and *Suc-Sacch* groups combined (*M* = 51.9%) and confirmed significantly higher preferences in the *Water-Sacch* group, *F*(1, 28) = 20.56, *p* < .001, η_p_^2^ = .42.

Inspection of Fig. 2A suggests a tendency for preferences to move towards 50% (i.e., indifference) from Test 1 to Test 2. We have noted this apparent result of repeating tests in previous experiments. Consequently, a one-way ANOVA was applied to preferences from Test 1 only. This also found a group effect, *F*(2, 28) = 11.01, *p* < .001, η_p_^2^ = .44, that was examined using the same planned contrasts. The difference between the *Sacch-Sacch* (*M* = 58.2%) and *Suc-Sacch* (*M* = 45.5%) groups was just significant, *F*(1, 28) = 4.41, *p* = .045, η_p_^2^ = .14, suggesting a very slight aversion to vanilla in the *Suc-Sacch* group. Higher preferences were again found in the *Water-Sacch* group (*M* = 74.8%) compared with the *Sacch-Sacch* and *Suc-Sacch* groups combined (*M* = 51.8%), *F*(1, 28) = 16.71, *p* < .001, η_p_^2^ = .37.

Prior to the saccharin preference test, the groups did not differ in intakes when given maltodextrin for the first time in preparation for the test that followed, *F* < 1. Preferences for unflavored saccharin over maltodextrin are shown in Fig. 2B. A mixed ANOVA applied to these data found a main effect of Test, *F*(1, 28) = 17.00, *p* < .001, η_p_^2^ = .38, but no effect of group or interaction (*F*s < 2.85). The planned comparison between preferences in the *Sacch-Sacch* group (*M* = 63.8%) and the *Suc-Sacch* (*M* = 50.6%) was significant, *F*(1, 28) = 4.76, *p* = .04, η_p_^2^ = .15, indicating indifference towards saccharin in the *Suc-Sacch* group, when averaged across both tests. No difference was detected between the *Water-Sacch* group (*M* = 59.8%) and the *Sacch-Sacch* and *Suc-Sacch* groups combined (*M* = 57.2%), *F* < 1.

A one-way ANOVA of preferences from Test 1 alone did find a group effect, *F*(2, 28) = 4.72, *p* = .02, η_p_^2^ = .25. The planned contrast between the *Sacch-Sacch* (*M* = 59.1%) and the *Suc-Sacch* (*M* = 41.0%) groups was significant, *F*(1, 28) = 7.49, *p* = .01, η_p_^2^ = .21, indicating a saccharin aversion in the *Sucrose* group. The comparison between the *Water-Sacch* group (*M* = 57.7%) and the *Sacch-Sacch* and *Suc-Sacch* groups combined (*M* = 50.0%) was not significant, *p* = .22.

## Discussion

The main results from this experiment were as follows. In Stage 2, acceptance of the saccharin solution in rats given sucrose in Stage 1 was reduced. Furthermore, this reduction in saccharin acceptance persisted throughout Stage 2, although the difference in saccharin intakes between the *Suc-Sacch* and *Sacch-Sacch* groups became smaller towards the end of Stage 2. As for the final preference tests, data analyses over both pairs of tests provided unclear results, with the exception of a robust vanilla preference in the *Water-Sacch* group. It appears that group differences were fragile and declined by the second test in each pair. On the other hand, outcomes were clearer when separate analyses were applied to data from the first of each pair of tests. For the *Suc-Sacch* group, these first tests revealed reduced preferences for both vanilla and saccharin, as consistent with the prediction of acquired aversions due to the switch from sucrose in Stage 1 to saccharin in Stage 2.

## Experiment 2

In the previous experiment rats that had received sucrose in Stage 1 experienced two kinds of flavor for the first time at the start of Stage 2—namely, that of saccharin and that of vanilla. There are at least two potential reasons why in Experiment 1 the predicted aversion to vanilla in the *Suc-Sacch* group was, at best, weak and transient. First, vanilla may have been overshadowed to some degree by the taste of saccharin; as seen in Fig. 2B, an aversion to saccharin that was also weak and transient was detected. Second, several studies using lithium-induced malaise have found that flavors (i.e., retronasal odors) are weaker cues (CSs) for association with the subsequent malaise than tastes (e.g., Durlach & Rescorla, 1980; Rusiniak et al., 1979, 1982). For both reasons, the present experiment used a saline solution (NaCl) to provide the target salty taste.

Another change in procedure was to limit the daily access to solutions to 30 min. This was to avoid a problem observed with some rats in Experiment 1, in which sessions of 1–2 h were given: After drinking their fill, these rats would rattle their bottles, thus causing some leakage and unreliable intake data for five rats, as noted in the Results section for Experiment 1. Since this problem arose mainly with the steel cages, these were not used in the present experiment. Using only the acrylic drinking chambers meant that two daily 30-min sessions were run. In light of apparent changes from the first to the second preference test seen in Experiment 1, the procedure used in this experiment was one in which, during a single test session, the position of the bottles was exchanged within the session.

As shown in Table 1B, a major change in design from the previous experiments was to include only two groups. As the main effect of interest was a predicted taste aversion in the *Suc-Sacch* group produced by a switch from sucrose to saccharin, a water control was not necessary and instead only an unshifted control, *Sacch-Sacch*, was included. A second change was to include a saline extinction phase after the initial preference tests. This was followed by a final NaCl preference test with the aim of assessing the extent to which any conditioned aversion to NaCl had extinguished.

### Method

#### Subjects

Twenty-four female Sprague-Dawley rats were purchased from ARC Perth. They were eight weeks old on arrival, when they were group housed under the same conditions as in Experiment 1. Experimental procedures began four weeks after their arrival. Throughout the experiment, unrestricted access to chow was available in the home cages. Except where noted below for the final test stage, water was also freely available until removed 3 h prior to each experimental session. Average weight at the start of the experiment was 275 g (range: 227–329 g).

#### Apparatus and solutions

All experimental sessions took place in twelve of the acrylic drinking chambers described for Experiment 1. The solutions included the 10% sucrose, 0.4% saccharin, and 4% maltodextrin solutions that were previously described. In addition, 0.5% (w/v) NaCl (commercial table salt) was added to the saccharin solution on the first 2 days of Stage 2 and was used in the preference tests.

#### Procedure

There were six sessions per week starting at 1600 hr. All sessions lasted 30 min, except where noted. Twelve rats from three cages were run in each squad, with two cages of *Suc-Sacch* rats and one of *Sacch-Sacch* rats in the first squad, and two cages of *Sacch-Sacch* rats and one of *Suc-Sacch* rats in the second.

For the first three sessions, all rats were given water to habituate them to drinking in the chambers. After the third day, they were allocated to two groups (*n* = 12), matched for body weight. In each of the twelve sessions in Stage 1 the *Suc-Sacch* rats were given the sucrose solution and the *Sacch-Sacch* rats the saccharin solution. In the first two sessions of Stage 2 (Days 13 and 14), all rats were given the compound solution of NaCl and saccharin. In the subsequent 10 sessions they were given only the saccharin solution.

The preference testing stage began with two 15-min sessions of two-bottle training when both bottles contained water. To familiarize rats with the switch of bottle positions to be used in the test, after 5 min the bottles were withdrawn and quickly replaced in the same position. Intakes from left and right bottles were calculated to assess side preferences, as previously described; no rats showed a side preference. To ensure adequate fluid intake in the drinking chambers, during this testing stage home cage water bottles were removed at the end of the previous day and restored after the session.

In the single 15-min saline preference test session, rats were given a two-bottle choice between NaCl in water and water only, with the starting position of NaCl counterbalanced in each group. Five min into the test these bottle positions were reversed. The next day, rats were given 30-min one-bottle access to the maltodextrin solution. On the following day they were given a 15-min preference test between 0.4% saccharin and 4% maltodextrin, with bottle positions counterbalanced and switched 5 min into the test, as described for the NaCl test.

After 1 week of rest with 24-h access to chow and water in the home cages, five sessions of saline extinction were given. Each group was divided into two, matched for preference ratios in the NaCl test, thus forming four subgroups (*n* = 6 each). Two subgroups (*Suc-Extn* and *Sacch-Extn*) were given 30-min daily access to the 0.5% NaCl solution, while the other two (*Suc-Non* and *Sacch-Non*) were given water. The NaCl preference test was then repeated.

#### Data analysis

Fluid intakes of Stages 1 and 2 were averaged across days into three blocks and analyzed with 2 × (3), Group × Block mixed ANOVAs. Independent-samples *t* tests (*Suc-Sacch* vs*. Sacch-Sacch* groups) were applied to the averages of each block in Stage 2 and maltodextrin intake prior to the saccharin preference test. Preference ratios of the tests were analyzed with 2 × 2, Group × Extinction ANOVAs. A 2 × 2 × (2), Group × Extinction × Day mixed ANOVA was applied to NaCl+saccharin intakes of Days 13 and 14, and a 2 × 2 × (5), Group × Extinction × Session mixed ANOVA to intakes across the NaCl extinction sessions.

### Results

**Fig. 3:**
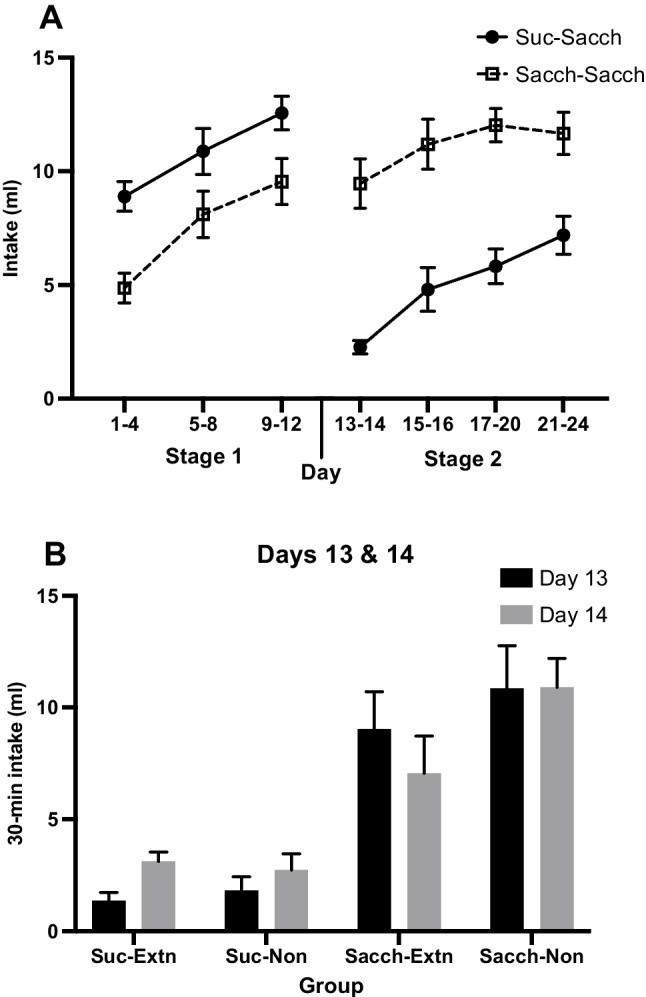
Experiment 2. **A** Mean intakes (±*SEM*) in Stage 1 of 10% sucrose and 0.4% saccharin solutions and in Stage 2 of the saccharin solution by both groups (*n* = 12). Access to the solutions was limited to 30 min daily in both stages. On Days 13–14, 0.5% NaCl was added to the saccharin solution for both groups. **B** Mean intake (+*SEM*) of the compound NaCl and saccharin solution in the first two sessions of Stage 2 (Days 13–14). NB, the NaCl extinction phase had not yet been introduced but group divisions are presented for comparison

Solution intakes, averaged over blocks, are shown for both stages in Fig. 3A. It can be seen that, as in the previous experiment, intakes of sucrose in Stage 1 were larger than intakes of saccharin and that both increased over the 12 sessions. This description was confirmed by a mixed ANOVA that found a main effect of group, *F*(1, 22) = 8.76, *p* = .007, η_p_^2^ = .29, and of block, *F*(2, 44) = 43.13, *p* < .001, η_p_^2^ = .66, with no interaction, *F* < 1.

On the first two days of Stage 2 (Days 13–14), intakes of the compound solution of NaCl and saccharin are shown in Fig. 3B. The groups were split into their allocated condition during the subsequent extinction stage. As predicted from previous results, rats given sucrose in Stage 1 (*Suc-Extn* and *Suc-Non*) drank far less of this solution than rats given saccharin in Stage 1 (*Sacch-Extn* and *Sacch-Non*). This group effect was confirmed by a mixed ANOVA, *F*(1, 20) = 43.95, *p* < .001, η_p_^2^ = .69, with no other main effects nor interactions, *p*s > .05.

Figure 3A shows intakes for the remaining sessions of Stage 2 (Days 15–24), when all rats received access to saccharin only. It may be seen that, as previously, intakes by the *Suc-Sacch* group remained lower than those by the *Sacch-Sacch* group, with intakes increasing over this stage somewhat more rapidly in the *Suc-Sacch* group. These data were analyzed by a mixed ANOVA, where, in the first block, intakes were averaged over Days 15 and 16 and in the next two blocks were averaged over four successive sessions. The analysis confirmed a main effect of group, *F*(1, 22) = 24.00, *p* < .001, η_p_^2^ = .52, and of block, *F*(1.5, 33.2) = 5.55, *p* = .014, η_p_^2^ = .20, and an interaction that was not quite significant (*p* = .08). Separate *t* tests applied to each block found a difference in intakes between the two groups in all three blocks, *t*s > 3.58.

**Fig. 4:**
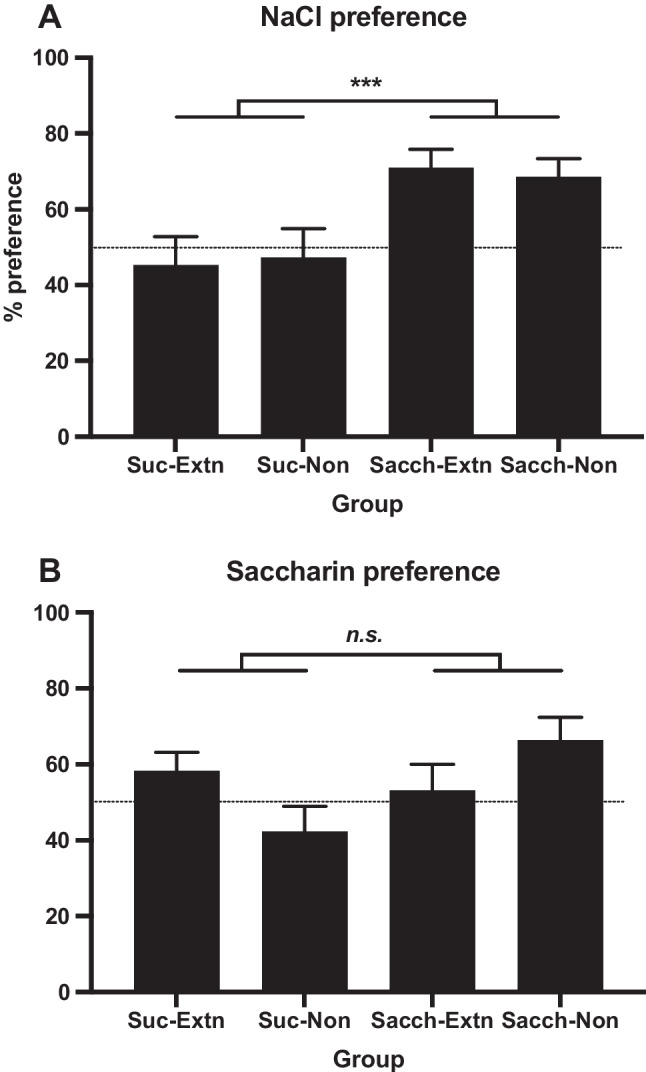
Experiment 2. Mean preference ratios (+*SEM*) in two-bottle choice tests between: **A** 0.5% NaCl solution and water; **B** 0.4% saccharin and 4% maltodextrin. *Note.* The NaCl extinction phase had not yet occurred, but group divisions are presented for comparison. *n.s.* = not significant; ***p = .001

Data from the two preference tests following Stage 2 (i.e., prior to the extinction stage) are shown in Fig. 4. The key finding from the NaCl test was that preferences for this solution were lower in the *Suc-Extn+Suc-Non* groups combined (*M* = 46.3%) than in the *Sacch-Extn+Sacch-Non* groups (*M* = 69.8%). The analysis found a main effect of group, *F*(1, 20) = 13.67, *p* = .001, η_p_^2^ = .41, with no effect of extinction or interaction, *F*s < 1 (see Fig. 4A). Since this test preceded the extinction procedure, the lack of effect of the extinction factor simply confirms that the groups were well-matched.

During familiarization to maltodextrin prior to the saccharin test, intakes did not differ between the *Suc-Sacch* and *Sacch-Sacch* groups, *t* < 1. In the test for saccharin preference, relative to maltodextrin, no difference in preference was found between the *Suc-Extn+Suc-Non* groups combined (*M* = 50.3%) and the *Sacch-Extn+Sacch-Non* groups (*M* = 59.7%). The analysis found no main effects (*p*s > .10); however, there was a Group × Extinction interaction, *F*(1, 20) = 5.73, *p* = .03, η_p_^2^ = .22 (see Fig. 4B). It is unclear what this interaction indicates, since the extinction factor is based on the conditions that the rats are about to experience.

**Fig. 5:**
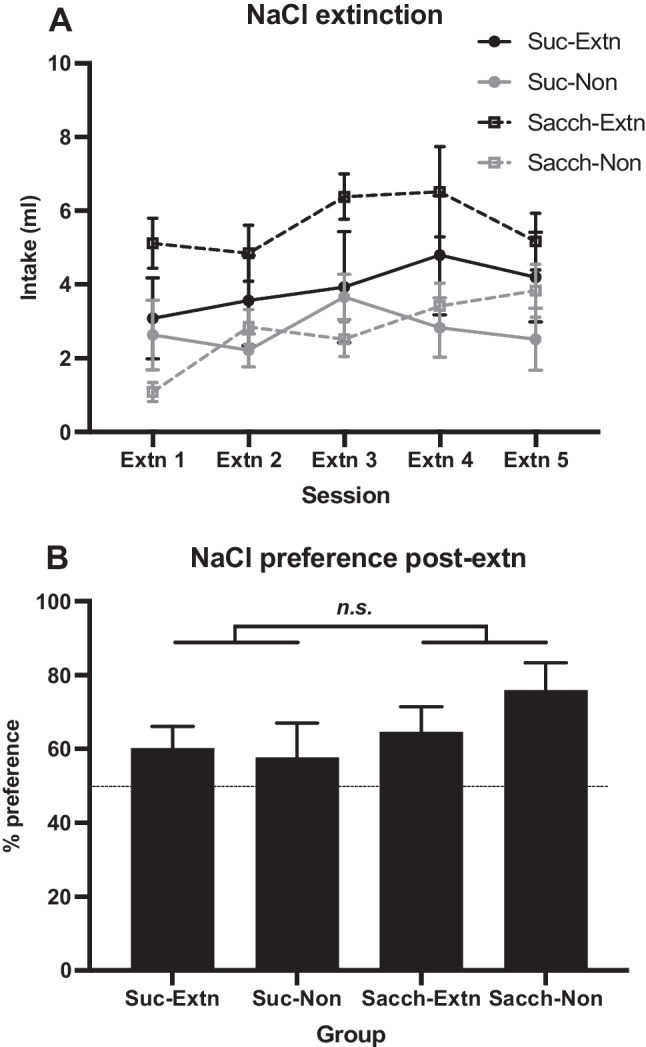
**A** Mean intake (±*SEM*) of 0.5% NaCl solution in the *Suc-Extn* and *Sacch-Extn* groups and water in the *Suc-Non* and *Sacch-Non* groups. **B** Mean preference ratios (+*SEM*) in the two-bottle choice test between 0.5% NaCl solution and water. *n.s.* = not significant

As seen in Fig. 5A, intakes of NaCl in the *Suc-Extn* and *Sacch-Extn* subgroups remained relatively constant across the extinction phase. A mixed ANOVA found a main effect of extinction, *F*(1, 20) = 8.71, *p* = .01, η_p_^2^ = .30, confirming higher intakes of NaCl in the extinction groups than of water in the nonextinction groups, and a linear trend across sessions, *F*(1, 20) = 6.27, *p* = .02, η_p_^2^ = .24, indicating increasing intakes across sessions, but no main effect of Stage 1 condition (sucrose vs. saccharin) and no interactions, *p*s >.10. Thus, although there was some suggestion in the final session that saline intakes of the *Suc-Extn* and *Sacch-Extn* groups were converging, there was no statistical support for such evidence of extinction of the saline aversion in the *Suc-Extn* group.

NaCl preference ratios post-extinction are shown in Fig. 5B; the analysis found no effect of group, extinction, or interaction, *p*s > .10.

### Discussion

The major new result from this experiment was the lower preference for the saline solution in the *Suc-Sacch* group that had previously experienced NaCl mixed with saccharin in the first two sessions of Stage 2. This result could represent either the acquisition of an aversion to saline or the failure to develop a preference for this taste that could have resulted from compounding it with saccharin in two sessions. It is notable that no reduced saccharin preference was detected in this experiment (see Fig. 4B). This may be because on Days 13 and 14 the taste of salt had overshadowed that of saccharin.

The group difference in the first NaCl preference test was not evident in the acceptance of NaCl during extinction sessions. Although the *Sacch-Extn* group drank slightly more of the saline solution during this phase, the group difference was not significant. Low power (only *n* = 6 per group) and high variability likely contributed to this outcome. Nonetheless, preferences in both *Suc-Sacch* subgroups had increased post-extinction regardless of whether they had been exposed to NaCl or water. This suggests that the apparent aversion to saline in the preextinction test (see Fig. 4A) was weak and transient.

## Experiment 3

The two previous experiments included a sucrose-to-saccharin transition from Stage 1 to Stage 2. In Experiment 2, Stage 2 started with salt added to the saccharin solution for two sessions, followed by 10 saccharin-only sessions. Although subsequent tests suggested an aversion to saline, the effect was weak and ambiguous. Two factors may have contributed to the relatively weak saline aversion found in Experiment 2. First, providing a NaCl + saccharin compound for the first two sessions of Stage 2 could have led to the taste of saccharin somewhat overshadowing the conditioning of a saline aversion. Second, continued exposure to saccharin for the remaining 10 sessions of Stage 2 may have weakened an aversion to saline via a within-compound association, saline-saccharin, acquired during the first two sessions of Stage 2. Therefore, in Experiment 3, saccharin was not included. Partly because saccharin appears to have a bitter component to its taste for rats, as it does for humans (e.g., Dess, 1993), the target taste used in this final experiment was provided by a weak quinine solution. The quinine concentration was chosen not to be too aversive for control rats switched from water but strong enough that it produced a downward shift in rats switched from a sucrose solution.

In the context of further testing for a SNC effect based on acquisition of a persistent taste aversion, the main aims of the experiment were (1) to establish a quinine aversion in rats that had received extended exposure to sucrose in Stage 1, relative to controls that had received only water, and (2) to test for extinction of this aversion in rats switched from sucrose to quinine in Stage 3 (*Suc-Quin* condition) relative to those switched from water to quinine (*Water-Quin* condition). As shown in Table 1C, in a 2 × 2 design, one factor, exposure, was whether rats were given 10% sucrose or water in Stage 1, the other, extinction, was whether they were given a weak quinine solution or water in Stage 3. In Stage 2, all rats received two sessions of the quinine solution, followed by a quinine versus water preference test. The four groups were named *Suc-Quin*, *Suc-Water*, *Water-Quin* and *Water-Water*.

### Method

#### Subjects

Thirty-two female Sprague-Dawley rats had previously served in an appetitive Pavlovian conditioning study in which they received food pellets and exposure to visual and auditory cues; they had no previous exposure to either sucrose or quinine. The rats were group housed under the same conditions as in Experiments 1 and 2. They were 17 weeks old at the start of the experiment with a mean weight of 267 g (range: 224–315 g). Unrestricted access to chow was given in the home cages. To ensure adequate intake of experimental fluids, rats were maintained on water restriction, as noted below. One rat from the *Suc-Quin* group was removed from the experiment due to excessive weight loss towards the end of Stage 3 and its data were excluded from all analyses.

#### Apparatus and solutions

The 16 drinking chambers were the acrylic drinking cages previously described. Solutions were 10% sucrose and a 12 mg/L solution (.00003 M) of quinine sulfate monohydrate (Aldrich Chemical Company). The quinine concentration was chosen to moderately suppress drinking; similar concentrations have suppressed drinking in rats to approximately 75%–80% of baseline water intake (Dess et al., 1988; Kiefer & Grijalva, 1980). Both of these studies used quinine hydrochloride, containing the same quinine content per mol as the quinine sulfate monohydrate used here. All solutions were mixed in tap water.

#### Procedure

There were six sessions per week, starting at 1300 hr. Sessions lasted 30 min, except where noted. Four cages (16 rats) were run in each squad, with one cage from each of the conditions. Initially, water was freely available in the home cages except for 3 h prior to each session. As water intakes for the first 9 days of Stage 1 were low, fluid restriction was then increased for the remainder of the experiment to 1 h daily, with 45-min access to water in home cages following each 15-min session and 60-min access on the one day each week when no experimental session was held.

All rats received three water pre-training sessions in the chambers, after which they were allocated to two weight-matched groups. Stage 1 comprised 12 sessions (Days 1-12) in which half the rats were given the sucrose solution and the other half were given water. In the first two sessions of Stage 2 (Days 13–14), all rats received 30-min exposure to the quinine solution; then two 15-min sessions of two-bottle water training (Days 15–16) were followed by the first quinine preference test (Day 17). As in Experiment 2, during two-bottle training rats were familiarized with the switch of bottles used in the test and side preferences were assessed; no side preferences were found. In the test, rats were given a choice between the quinine solution and water, with the starting position of quinine counterbalanced and bottle positions reversed after 5 min, as previously described.

For Stage, 3 half of each group was allocated to either the *Quin* or *Water* conditions, matching for quinine preferences in the previous test. Stage 3 comprised 12 sessions (Days 18–29) of the quinine solution for the *Suc-Quin* and *Water-Quin* groups and water for the *Suc-Water* and *Water-Water* groups. Following two further two-bottle sessions of water training, a second quinine preference test was carried out.

#### Data analysis

Fluid intakes of Stage 1 exposure and Stage 3 extinction were averaged across sessions into three four-session blocks and analyzed with 2 × 2 × (3), Exposure × Extinction × Block mixed ANOVAs. (Allocation to Stage 3 fluids had not yet taken place in Stage 1.) Separate 2 × 2, Exposure × Extinction ANOVAs were applied to averages of each block in Stage 3 and to preference ratios of the tests; simple effects analyses examined group differences of interest. Stage 2 intakes of quinine (Days 13–14) were examined with a 2 × 2 × (2), Exposure × Extinction × Day mixed ANOVA.

### Results

**Fig. 6:**
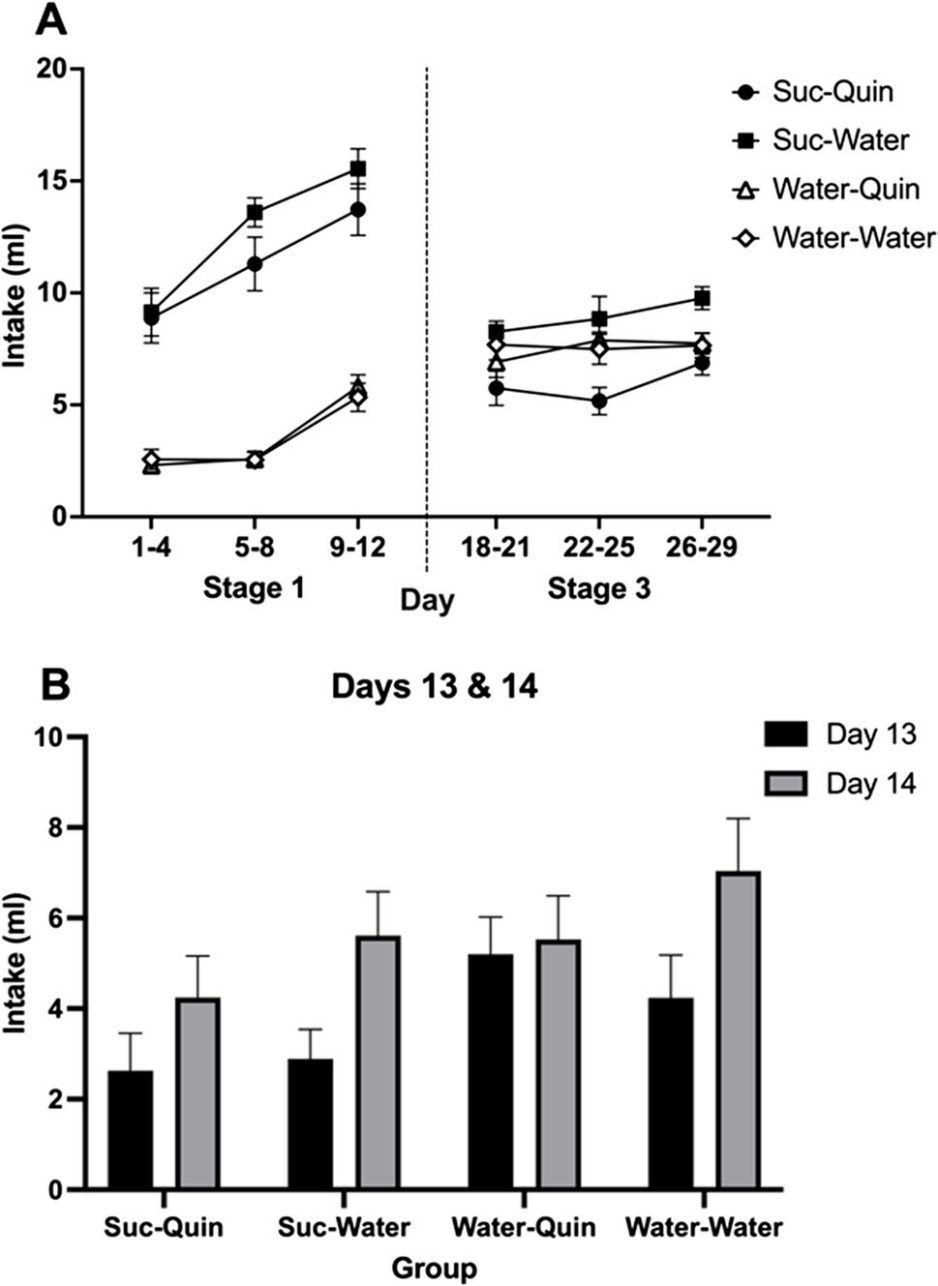
Experiment 3. **A** Mean intakes (±*SEM*) in Stage 1 of 10% sucrose vs. water and in Stage 3 of quinine solution vs. water. **B** Mean intake (+*SEM*) of quinine solution in the first two sessions of Stage 2 (Days 13–14; note, groups were not yet divided into the Stage 3 quinine vs. water conditions)

Intakes of solutions in Stages 1 and 3, averaged over blocks, are shown in Fig. 6A. The 2 × 2 × (3) ANOVA applied to Stage 1 intakes confirmed higher intakes of sucrose than of water, with a main effect of exposure, *F*(1, 27) = 173.21, *p* < .001, η_p_^2^ = .87, and of block, *F*(2, 54) = 77.37, *p* < .001, η_p_^2^ = .74, and an Exposure × Block interaction, *F*(2, 54) = 11.81, *p* < .001, η_p_^2^ = .30. As suggested by Fig. 6A, these interactions confirm different patterns of increasing intake for sucrose and water.

Figure 6B shows quinine intakes in the first two sessions of Stage 2 (Days 13–14). The mixed analysis found a main effect of exposure, *F*(1, 27) = 4.40, *p* = .045, η_p_^2^ = .14, with no main effect or interaction involving extinction, *F*s < 1. This confirmed that intakes of quinine in the *Suc* groups combined (*M* = 7.7 ml) were lower than in the *Water* groups (*M* = 11.0 ml). There was also an effect of day, *F*(1, 27) = 15.03, *p* = .001, η_p_^2^ = .36, indicating the loss of neophobia and a greater acceptance of quinine on Day 14. No interactions involving day were found, *p*s > .05.

**Fig. 7:**
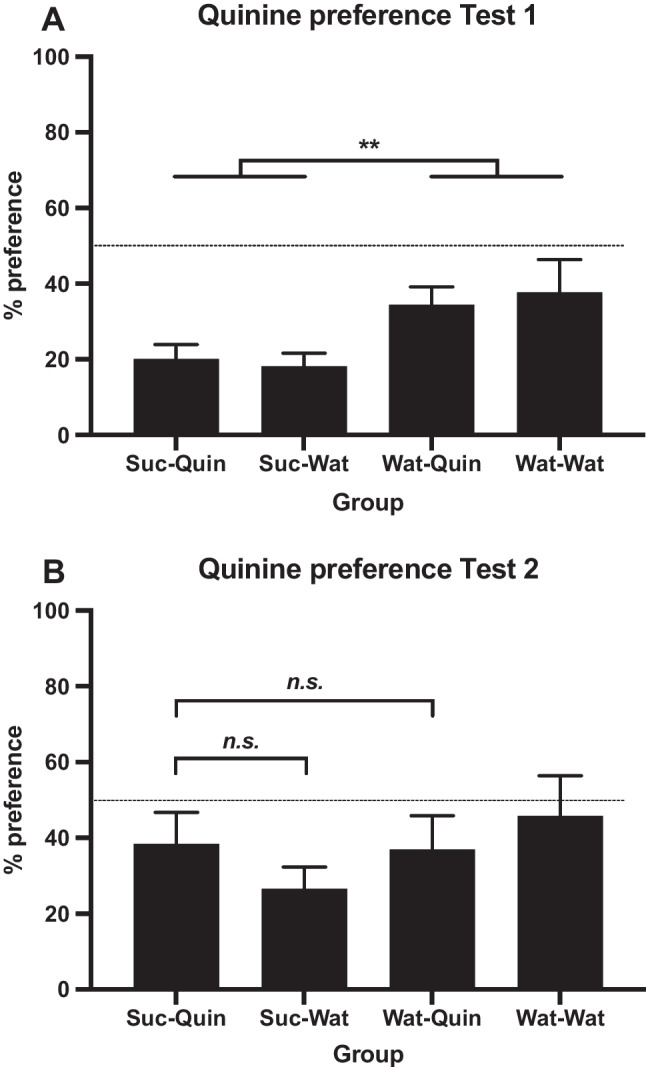
Mean quinine preferences (+*SEM*) presented as percentage ratio of quinine versus water intake at two-bottle choice tests. **A** Test 1 after two sessions (Days 13–14) of quinine exposure (allocation to Stage 3 fluids had not yet occurred), **p = .006. **B** Test 2 after Stage 3 quinine or water intake, *n.s.* = not significant (simple effects)

Figure 7A shows quinine preferences in the first quinine vs. water choice test. The analysis found a main effect of exposure (sucrose vs. water), *F*(1, 27) = 8.94, *p* = .006, η_p_^2^ = .25. Effective matching of groups prior to the extinction stage was confirmed by the absence of a main effect of extinction or interaction, *F*s < 1. Mean preference ratios were below 50% in all groups but those that had received sucrose in Stage 1 had significantly lower quinine preferences on average (*M* = 18.5%) than those that had received water (*M* = 36.1%). This indicates that the *Suc* groups demonstrated an aversion to the taste of quinine that was greater than the innate aversion to quinine evident in the *Water* control groups.

Stage 3 intakes of quinine vs. water are shown on the right of Fig. 6A. The mixed analysis found no main effect of exposure, *F* < 1. There was a main effect of extinction (quinine vs. water), *F*(1, 27) = 10.01, *p* = .004, η_p_^2^ = .27, and of block, *F*(2, 54) = 3.49, *p* = .038, η_p_^2^ = .11, and an interaction between the stages, *F*(1, 27) = 8.80, *p* = .006, η_p_^2^ = .25. A 2 × 2 applied to the first block found only a significant effect of extinction, *F*(1, 27) = 6.36, *p* = .02, η_p_^2^ = .19, with less quinine consumed than water. Analysis of the second block revealed the same main effect, *F*(1, 27) = 5.37, *p* = .03, η_p_^2^ = .17, and an interaction between stages, *F*(1, 27) = 8.36, *p* = .01, η_p_^2^ = .24. The simple effects comparison of *Suc-Quin* versus *Water-Quin* was only significant in the second block, *F*(1, 27) = 7.18, *p* = .01, η_p_^2^ = .21, with lower quinine intake in the *Suc-Quin* group. In the third block there was also a main effect of extinction, *F*(1, 27) = 7.40, *p* = .01, η_p_^2^ = .22, and an interaction, *F*(1, 27) = 8.35, *p* = .01, η_p_^2^ = .24. In all of the Stage 3 blocks, the *Suc-Quin* versus *Suc-Water* simple effect was significant (smallest *F* = 7.14), indicating the groups exposed to sucrose in Stage 1 consistently drank less quinine than water in Stage 3.

Quinine preferences in Test 2 are shown in Fig. 7B. The analysis found no significant effects, *p*s > .10; the effect of Exposure (sucrose vs. water) evident in Test 1 was no longer present. Specifically addressing our second aim—whether the quinine aversion in the *Suc-Quin* group would extinguish—preferences in the *Suc-Quin* group increased after Stage 3 exposure to quinine (Test 1 *M* = 20.1%, Test 2 *M* = 38.4%) and no longer differed (*F* < 1) from preferences in the *Water-Quin* control group (Test 1 *M* = 34.5%, Test 2 *M* = 36.9%), indicating the quinine aversion had extinguished. A simple effects analysis also compared preferences in the *Suc-Quin* group (*M* = 38.4%) with the *Suc-Water* (*M* = 26.6%), the difference was not significant, *F* < 1.

### Discussion

This experiment achieved its two aims. The first was to establish an aversion to quinine in rats that had previously been given the sucrose solution that was greater than that shown by rats previously given water. This was shown both by the greater avoidance of quinine (with mean preference of 18.5% relative to water) in the first quinine test by the *Suc* rats than by the *Water* rats (mean preference of 36.1%), as shown in Fig. 7A, and in Stage 3 by the lower acceptance of quinine by the *Suc-Quin* than by the *Water-Quin* rats, as shown in Fig. 6A.

The second aim was to extinguish this aversion. This was achieved by giving the *Suc-Quin* group the quinine solution for twelve sessions, after which their preference for quinine was no different from that of the *Water-Quin* group, and greater than that of the *Suc-Water* group, as shown in Fig. 7B.

This conditioning and then extinction of a quinine aversion was superimposed upon the innate—or at least, preexisting—aversion to quinine that rats and most other mammals display when encountering its taste. This aversion was displayed when the present rats were first exposed to the concentration of quinine used here but, with repeated exposure, the rats showed a reduction in their aversion; see Fig. 6B and the absence of any avoidance of this solution shown by the *Water-Water* group in Fig. 7B.

## General discussion

The main aim of this study was to test the hypothesis that a qualitative successive negative contrast effect—one that introduces a new taste during the downshift from a highly valued incentive to a much less valued incentive—involves the conditioning of an aversion to the new taste. These three experiments provided increasingly strong evidence in support of this hypothesis. The finding in Experiment 3 of the clearest evidence of taste aversion learning—in this case, as a result of a downshift from sucrose to quinine—was almost certainly due to the absence of any potentially competing taste following the downshift. In the first two experiments, the presence of saccharin seems to have partially overshadowed the acquisition of an aversion to the vanilla flavor used in Experiment 1 and the salty taste used in Experiment 2.

In the majority of consummatory SNC experiments that have employed the 32% sucrose to 4% sucrose downshift, the rats have been food restricted. The importance of animals’ motivational state in the degree to which they show a contrast effect, when downshifted from 32% to 4% sucrose, has been shown in a series of experiments by Ruiz-Salas et al. (2020). They found that the contrast effect was greatest when their rats were food restricted and not detectable when water deprived or given food prior to postshift sessions. The possible effects of the motivational state were not examined in the series of experiments reported here; these rats were neither food nor fluid restricted before or following the shift. Mild fluid restriction was employed only during testing stages. It seems plausible that the apparent contrast effects we have studied would not have been as persistent if the rats had been food restricted. Although this possibility was not investigated in the present study, the question of whether the persistence of a qualitative contrast effect depends on the animals’ motivational state could be usefully investigated in future studies.

A general limitation of the present study is its reliance on data from preference tests. Two kinds of problems arise from the use of such tests. First, when groups are compared on their choice of some target flavor or taste (e.g., saccharin in Experiments 1 and 2) with another taste solution (e.g., maltodextrin in those first two experiments), it is possible that the experimental manipulation also affected an animal’s response to the supposedly neutral comparison solution. We recently found such an example. In the experiment reported by Rehn et al. (2023), a number of groups were tested for liking for a saccharin solution in a two-bottle test that pitted this solution against a sucrose solution. Only the rats previously exposed to sucrose showed a preference for this solution; thus, this did not serve as a ‘neutral’ stimulus against which to assess liking for saccharin.

The second limitation to the use of preference tests to assess whether an aversion has been acquired to some target solution is that it cannot reveal anything about the nature of the aversion. This is where examining the microstructure of licking patterns can be useful in indicating whether the change in response to a solution reflects a reduction in liking, as in Rehn et al. (2023). Furthermore, oral taste reactivity tests can reveal whether a reduction in intake of a target solution involves the development of a disgust reaction to the target (e.g., Grill & Norgren, 1978; López et al., 2023).

The present evidence using preference tests complements the results from our parallel study using lick cluster size as the main outcome measure (Rehn et al., 2023). The results from the extinction stages in the present Experiments 2 and 3 suggest that repeated exposure to the averted tastes can rapidly extinguish the aversion. This is also consistent with the evidence from lick cluster measurements in Rehn et al. (2023). However, both sets of results leave unanswered the question that prompted this series of experiments—namely, why did the transition from 10% sucrose to a normally acceptable saccharin solution lead to low intakes of the saccharin solutions, ones that failed to increase over several weeks (Kendig et al., 2018)?

Our only tentative answer to this question to date contains two elements. The first is that a second kind of associative learning is important—namely, between approach responses to the drinking spout and the frustration experienced when the unexpected downshift occurred. We assume that the resulting reduction of approach responses is slower to extinguish than the conditioned taste aversion, either because the latter is more weakly conditioned by frustration or because avoidance (as the opposite of the punished approach) is in effect self-reinforcing. This proposal resembles the account proposed by Dwyer (2009) to explain the persistence in extinction of conditioned taste aversions beyond the point at which the hedonic value of the taste has recovered.

The second element addresses the question of why the transition from sucrose to saccharin produces a particularly persistent reduction in intakes. Both sucrose and saccharin have a sweet taste, which in the latter is combined with a bitter taste. We propose that in rats with immediately prior experience of a sweet taste the bitter element of saccharin is more prominent than in rats not recently exposed to a sweet taste. This results in a form of perceptual learning, whereby the unpleasant bitter component of saccharin remains more prominent for sweet-experienced rats.

Whatever the merits of the above suggestions, the clear conclusion from the present experiments is that in cases of a qualitative SNC an aversion is conditioned to the new taste that rats encounter during the downshift.

## References

[CR1] Boakes, R. A., Rehn, S., Badolato, C., & Rooney, K. B. (2020). Reduced acceptance of saccharin solutions by rats previously consuming more highly palatable solutions. *Physiology & Behavior,**218*, Article 112822. 10.1016/j.physbeh.2020.112822 32004547 10.1016/j.physbeh.2020.112822

[CR2] Crespi, L. P. (1942). Quantitative variation of incentive and performance in the white rat. *The American Journal of Psychology,**55*, 467–517. 10.2307/1417120

[CR3] Dess, N. K. (1993). Saccharin’s aversive taste in rats: Evidence and implications. *Neuroscience & Biobehavioral Reviews,**17*(4), 359–372. 8309647 10.1016/s0149-7634(05)80113-7

[CR4] Dess, N. K., Chapman, C. D., & Minor, T. R. (1988). Inescapable shock increases finickiness about drinking quinine-adulterated water in rats. *Learning and Motivation,**19*, 408–424. 10.1016/0023-9690(88)90048-3

[CR5] Durlach, P. J., & Rescorla, R. A. (1980). Potentiation rather than overshadowing in flavor-aversion learning: An analysis in terms of within-compound associations. *Journal of Experimental Psychology: Animal Behavior Processes,**6*, 175–187. 10.1037/0097-7403.6.2.175 7373231

[CR6] Dwyer, D. M. (2009). Short article: Microstructural analysis of ingestive behaviour reveals no contribution of palatability to the incomplete extinction of a conditioned taste aversion. *Quarterly Journal of Experimental Psychology,**62*(1), 9–17. 10.1080/17470210802215152 18622888

[CR7] Elliott, M. H. (1928). *The effect of change of reward on the maze performance of rats*. University of California Press.

[CR8] Flaherty, C. F. (1996). *Incentive relativity*. Cambridge University Press.

[CR9] Flaherty, C. F., & Largen, J. (1975). Within-subjects positive and negative contrast effects in rats. *Journal of Comparative and Physiological Psychology,**88*(2), 653. 1150943 10.1037/h0076416

[CR10] Flaherty, C. F., & Rowan, G. A. (1986). Successive, simultaneous, and anticipatory contrast in the consumption of saccharin solutions. *Journal of Experimental Psychology: Animal Behavior Processes,**12*(4), 381. 3772302

[CR11] Freidin, E., Cuello, M. I., & Kacelnik, A. (2009). Successive negative contrast in a bird: Starlings’ behaviour after unpredictable negative changes in food quality. *Animal Behaviour,**77*(4), 857–865.

[CR12] Grill, H. J., & Norgren, R. (1978). Taste reactvity test: 1. Mimetic responses togustatory stimuli in neurologically normal rats. *Brain Research,**143*, 263–279. 630409 10.1016/0006-8993(78)90568-1

[CR13] Kendig, M. D., Fu, M. X., Rehn, S., Martire, S. I., Boakes, R. A., & Rooney, K. B. (2018). Metabolic and cognitive improvement from switching to saccharin or water following chronic consumption by female rats of 10% sucrose solution. *Physiology & Behavior,**188*, 162–172. 29425973 10.1016/j.physbeh.2018.02.008

[CR14] Kiefer, S. W., & Grijalva, C. V. (1980). Taste reactivity in rats following lesions of the zona incerta or amygdala. *Physiology & Behavior,**25*, 549–554. 10.1016/0031-9384(80)90120-1 7208651 10.1016/0031-9384(80)90120-1

[CR15] López, M., Dwyer, D. M., Gasalla, P., Begega, A., & Jove, C. (2023). Odor-taste pairings lead to the acquisition of negative hedonic qualities by the odor in aversion learning. *Physiology & Behavior,**269*, Article 114269. 37328020 10.1016/j.physbeh.2023.114269

[CR16] Mitchell, C. P., & Flaherty, C. F. (2005). Differential effects of removing the glucose or saccharin components of a glucose–saccharin mixture in a successive negative contrast paradigm. *Physiology & Behavior,**84*(4), 579–583. 15811393 10.1016/j.physbeh.2005.02.005

[CR17] Papini, M. R. (2003). Comparative psychology of surprising nonreward. *Brain, Behavior and Evolution,**62*(2), 83–95. 12937347 10.1159/000072439

[CR18] Pellegrini, S., & Mustaca, A. E. (2000). Consummatory successive negative contrast with solid food. *Learning & Motivation,**31*, 200–209.

[CR19] Pellegrini, S., Muzio, R. N., Mustaca, A. E., & Papini, M. R. (2004). Successive negative contrast after partial reinforcement in the consummatory behavior of rats. *Learning and Motivation,**35*(4), 303–321.

[CR20] Ramirez, I. (1994). Glucose polymer taste is not unitary for rats. *Physiology & Behavior,**55*(2), 355–360. 8153178 10.1016/0031-9384(94)90146-5

[CR21] Rehn, S., Boakes, R. A., & Dwyer, D. (2023). Switching from sucrose to saccharin: Extended successive negative contrast is not maintained by hedonic changes. *Journal of Experimental Psychology: Animal Learning and Cognition,**49*, 289–295. 37883032 10.1037/xan0000362

[CR22] Ruiz-Salas, J. C., de la Casa, G., & Papini, M. R. (2020). Dimensions of sucrose solutions in the successive negative contrast effect. *Learning and Motivation,**69*, Article 101615.

[CR23] Rusiniak, K. W., Hankins, W. G., Garcia, J., & Brett, L. P. (1979). Flavor-illness aversions: Potentiation of odor by taste in rats. *Behavioral and Neural Biology,**25*(1), 1–17. 454334 10.1016/s0163-1047(79)90688-5

[CR24] Rusiniak, K. W., Palmerino, C. C., Rice, A. G., Forthman, D. L., & Garcia, J. (1982). Flavor-illness aversions: Potentiation of odor by taste with toxin but not shock in rats. *Journal of Comparative and Physiological Psychology,**96*, 527–539. 10.1037/h0077902 6288778 10.1037/h0077902

[CR25] Sclafani, A. (1991). Starch and sugar tastes in rodents: An update. *Brain Research Bulletin,**27*(3/4), 383–386. 1959034 10.1016/0361-9230(91)90129-8

